# Characteristic computed tomography features in mesenchymal-epithelial transition exon14 skipping-positive non-small cell lung cancer

**DOI:** 10.1186/s12890-022-02037-4

**Published:** 2022-06-30

**Authors:** Naokazu Watari, Kakuhiro Yamaguchi, Hiroaki Terada, Kosuke Hamai, Ken Masuda, Yoshifumi Nishimura, Shinjiro Sakamoto, Takeshi Masuda, Yasushi Horimasu, Shintaro Miyamoto, Taku Nakashima, Hiroshi Iwamoto, Hiroyasu Shoda, Nobuhisa Ishikawa, Kazunori Fujitaka, Kozue Miyazaki, Yoshihiro Miyata, Hironobu Hamada, Kazuo Awai, Noboru Hattori

**Affiliations:** 1 Department of Respiratory Medicine, Hiroshima University Hospital, 1-2-3 Kasumi, Minami-ku, Hiroshima, 734-8551 Japan; 2 Department of Internal Medicine, Mihara Medical Association Hospital, 1-15-1 Miyaura, Mihara, 723-0051 Japan; 3 Department of Diagnostic Radiology, Graduate School of Biomedical and Health Sciences, Hiroshima University, 1-2-3 Kasumi, Minami-ku, Hiroshima, 734-8551 Japan; 4 Department of Respiratory Medicine, Hiroshima Prefectural Hospital, 1-5-54 Ujina-Kanda, Minami-ku, Hiroshima, 734-8530 Japan; 5 Department of Respiratory Internal Medicine, Hiroshima City Hiroshima Citizens Hospital, 7-33 Motomachi, Naka-ku, Hiroshima, 730-8518 Japan; 6 Department of Respiratory Medicine, National Hospital Organization Higashihiroshima Medical Center, 513 Jike, Saijo-cho, Higashihiroshima, 739-0041 Japan; 7 Department of Surgical Oncology, Hiroshima University, 1-2-3 Kasumi, Minami-ku, Hiroshima, 734-8551 Japan

**Keywords:** Mesenchymal-epithelial transition exon14 skipping, Computed tomography, Non-small cell lung cancer, Driver gene mutation, Imaging examination

## Abstract

**Background:**

Mesenchymal-epithelial transition exon14 (METex14) skipping is one of the therapeutic driver oncogene mutations in non-small cell lung cancer (NSCLC), and can be treated with tepotinib and capmatinib. There is only one report on computed tomography (CT) findings of METex14 skipping-positive NSCLC, which shows that the primary tumor tends to have a large mass in the upper lobe, and extrathoracic metastases are common. This study examined the CT findings of METex14 skipping-positive NSCLC, focusing on the features of the margins and internal structures.

**Methods:**

We consecutively included patients with METex14 skipping-positive NSCLC who were diagnosed between January 2018 and December 2020 at four independent institutions. We retrospectively reviewed the patient demographics and CT findings for tumor margins (invasion into surrounding tissue, lobulation, pleural indentation, spicula, and ground-glass opacity) and internal structures (air bronchograms, cavitation and internal low-density area).

**Results:**

Fifteen patients with METex14 skipping-positive NSCLC were identified. Almost half of the patients were men (7/15; 46.7%), and their median age was 75.0 years. More than half were either current or former smokers (9/15; 60.0%). A vast majority of histological subtypes were adenocarcinoma (10/15; 66.7%), followed by pleomorphic carcinoma (3/15; 20.0%) and squamous cell carcinoma (2/15; 13.3%). With regard to CT findings, most primary tumors presented as masses larger than 30 mm (12/15; 80.0%) and were located in the upper lobes (12/15; 80.0%). Invasion into surrounding tissue and presence of internal low-density areas were observed in 60.0% (9/15) and 66.7% (10/15) of the primary tumors, respectively. Additionally, their frequencies increased to 72.7% (8/11) and 90.9% (10/11) in stage III/IV cases, respectively. In lymph node metastasis, internal low-density areas were observed in 8/10 cases (80.0%). Although these two CT features were rarely observed in distant metastases at diagnosis, they became apparent with progression of the metastatic tumor size.

**Conclusions:**

METex14 skipping-positive NSCLC tumors tend to invade surrounding tissue and possess internal low-density areas. These CT findings might be characteristic of METex14 skipping-positive NSCLC.

**Supplementary Information:**

The online version contains supplementary material available at 10.1186/s12890-022-02037-4.

## Introduction

The presence of driver gene mutations affects the treatment strategy and prognosis of non-small cell lung cancer (NSCLC) [1]. Mesenchymal-epithelial transition exon14 (METex14) skipping is a driver oncogene mutation for which molecular target drugs have been established. Tyrosine kinase inhibitors (TKIs) targeting METex14 skipping, such as tepotinib and capmatinib, have been shown to improve the prognosis of patients with METex14 skipping-positive NSCLC [2, 3]. The clinical characteristics of METex14 skipping-positive NSCLC are diverse; for example, the prevalence of METex14 skipping by histological subtypes was approximately 2–3% in adenocarcinoma, 2% in squamous cell carcinoma, and 20–30% in sarcomatoid carcinoma, including pleomorphic carcinoma [4–6]. Therefore, it is necessary to prescribe MET-TKIs to all patients with METex14 skipping-positive NSCLC through appropriate molecular testing of METex14 skipping.

Several studies have reported that characteristic computed tomography (CT) findings are useful for identifying patients with NSCLC harboring driver oncogene mutations. For example, NSCLC with epidermal growth factor receptor (EGFR) mutations is characterized by the convergence of surrounding structures, ground-glass opacity (GGO), and multiple intrapulmonary metastases [7–10]. NSCLC with anaplastic lymphoma kinase (ALK) rearrangement has been reported to show hypoattenuation in the primary tumor and extranodal invasion in lymph node metastasis [11, 12]. In CT findings of METex14 skipping-positive NSCLC, there is only one report that the primary tumor often appears in the upper/middle lobe (70.2%) and it is likely to be a mass larger than 30 mm (63.1%) [13]. However, there are no reports on the characteristics of the margins and internal structure of METex14 skipping-positive NSCLC.

The aim of this study was to examine the CT findings of METex14 skipping-positive NSCLC, focusing on the features of the margin and internal structure. Firstly, the CT findings of the primary tumor, lymph node metastasis, and distant metastasis at diagnosis were evaluated. Secondly, changes in CT findings of distant metastasis during cancer treatment were presented by considering the CT features identified at diagnosis.

## Methods

### Patient selection and patient background

We consecutively identified patients with METex14 skipping-positive NSCLC diagnosed between January 2018 and December 2020 at four institutions: Hiroshima Prefectural Hospital, Hiroshima City Hiroshima Citizens Hospital, Higashi Hiroshima Medical Center, and Hiroshima University Hospital. Using medical records, we retrospectively reviewed the patients’ background and clinicopathological findings, including age, sex, smoking history, histological types of lung cancer, and stage.

This study was conducted in accordance with the principles of the Declaration of Helsinki and approved by the Ethical Committee of Hiroshima University Hospital (no. E2319). Informed consent for use of the CT images and clinical data for research purposes was obtained by the opt-out method, which is approved by the Ethical Committee of Hiroshima University Hospital (no. E2319).

### Molecular testing

Molecular testing was performed on the tumor tissue or blood samples. The METex14 skipping status was determined using the Oncomine Dx Target Test Multi‐CDx System or ArcherMET.

### Imaging protocol and image analysis

All patients underwent CT prior to the initiation of cancer-specific treatment. Although contrast-enhanced CT scanning was performed based on the protocol defined in each institution, equilibrium phase CT images with a 5-mm reconstruction thickness could be evaluated for all cases where iodinated intravenous contrast medium was used. If there were no contrast-enhanced images, plain CT images with a 5-mm reconstruction thickness were evaluated. An experienced diagnostic radiologist and an experienced respiratory physician reviewed the CT images concurrently, and the CT findings were determined and recorded by consensus. The CT features of the primary lung tumor, lymph node metastases and distant metastases were also assessed.

The CT features of the primary tumor were assessed by considering size, lobar location, presence of invasion into surrounding tissue, lobulation, pleural indentation, spicula, GGO, air bronchograms, cavitation, and internal low-density areas. Invasion into the surrounding tissues was defined as direct invasion into adjacent organs. Lobulation was defined as an abrupt bulge in the lesion contour. A spicula was defined as a strand extending from the nodule margin into the lung parenchyma without reaching the pleural surface. GGO was defined as a hazy increase in attenuation that did not obscure the normal lung markings. Air bronchograms were defined as air-filled bronchi that appeared as radiolucent branching bands within pulmonary densities. Cavitation was indicated by the presence of a round or oval air density in the tumor, with a relatively thick wall. An internal low-density area was defined as an area inside the tumor that showed low uptake relative to the surrounding tumor parenchyma in contrast-enhanced and/or plain CT. Lymph nodes measuring > 10 mm on the short axis were considered malignant. The presence of metastases in the lungs, pleura, brain, adrenal gland, liver, stomach, spleen, bones, and soft tissue was documented. Pulmonary lymphangitic carcinomatosis, pleural metastasis, and pericardial metastasis were also documented. The CT features of lymph nodes and distant metastases were also assessed by focusing on the identified features of the primary tumor.

### Statistical analysis

Values are expressed as median and interquartile range. When differences among groups were examined, Fisher's exact test was used to compare the nominal variables. Statistical significance was set at *p* < 0.05. All statistical analyses were performed using EZR (Saitama Medical Center, Jichi Medical University, Saitama, Japan) [14], a graphical user interface for R (The R Foundation for Statistical Computing, Vienna, Austria).

## Results

### Clinicopathologic characteristics

Fifteen METex14 skipping-positive NSCLC patients were identified (Additional file 1: Table S1). Seven cases were identified by the Oncomine Dx Target Test Multi-CDx System only, while the other eight cases were identified by ArcherMET. Clinicopathological characteristics of the patients are summarized in Table 1. Almost half of the patients were male (7/15; 46.7%), and their median age was 75.0 years. More than half were either current or former smokers (9/15; 60.0%). A vast majority of histological subtypes were adenocarcinoma (10/15; 66.7%), followed by pleomorphic carcinoma (3/15; 20.0%), and squamous cell carcinoma (2/15; 13.3%). There were more cases of stage III/IV NSCLC than of stage I/II NSCLC.

**Table 1:** Clinicopathological characteristics of METex14 skipping-positive NSCLC patients

Clinicopathologic characteristics of patients with METex14 skipping-positive NSCLC	
**Subjects, n**	15
**Age, years**	75.0 (71.0–84.0)
**Sex**	
Male, n (%)	7 (46.7)
**Smoking history**	
Current/former, n (%)	9 (60.0)
Never, n (%)	6 (40.0)
**Histology**	
Adenocarcinoma, n (%)	10 (66.7)
Pleomorphic carcinoma, n (%)	3 (20.0)
Squamous cell carcinoma, n (%)	2 (13.3)
**Stage, I/II/III/IV**	3/1/5/6

Data are presented as medians and interquartile ranges

### CT imaging features of the primary tumor

The CT imaging features of the primary tumor with METex14 skipping are summarized in Table 2. Three patients with Stage I/II NSCLC were evaluated only by plain CT (Additional file 1: Figure S1).

**Table 2:** CT imaging features of the primary tumor in METex14 skipping-positive NSCLC

	All patients	Stage I/II	Stage III/IV
**Subjects, n**	15	4	11
**Size**			
Median (mm)	45.0 (33.0–70.0)	31.5 (26.0–37.5)	65.0 (37.5–77.5)
Mass (> 30 mm), n (%)	12 (80.0)	2 (50.0)	10 (90.9)
Nodule (≤ 30 mm), n (%)	3 (20.0)	2 (50.0)	1 (9.1)
**Lobar location**			
Upper lobe/upper segment, n (%)	12 (80.0)	3 (75.0)	9 (81.8)
Middle lobe/lingular segment, n (%)	0 (0.0)	0 (0.0)	0 (0.0)
Lower lobe, n (%)	3 (20.0)	1 (25.0)	2 (18.2)
**Margin**			
Invasion into surrounding tissue, n (%)	9 (60.0)	1 (25.0)	8 (72.7)
Lobulation, n (%)	2 (13.3)	0 (0.0)	2 (18.2)
Pleural indentation, n (%)	3 (20.0)	1 (25.0)	2 (18.2)
Spicula, n (%)	3 (20.0)	1 (25.0)	2 (18.2)
Ground-glass opacity, n (%)	1 (6.7)	1 (25.0)	0 (0.0)
**Internal structure**			
Air bronchograms, n (%)	2 (13.3)	1 (25.0)	1 (9.1)
Cavitation, n (%)	2 (13.3)	0 (0.0)	2 (18.2)
Internal low-density area, n (%)	10 (66.7)	0 (0.0)	10 (90.9)

Data are presented as medians and interquartile ranges

Most tumors presented as masses larger than 30 mm (12/15; 80.0%), and their median size was 45.0 mm (33.0–77.0 mm). They were predominantly located in the upper lobes (12/15; 80.0%). The primary tumors invaded into surrounding tissue and possessed an internal low-density area in 60.0% (9/15) and 66.7% (10/15) of cases, respectively. Lobulation, pleural indentation, spicula, air bronchograms, cavitation, and GGO were rarely observed.

**Fig. 1:**
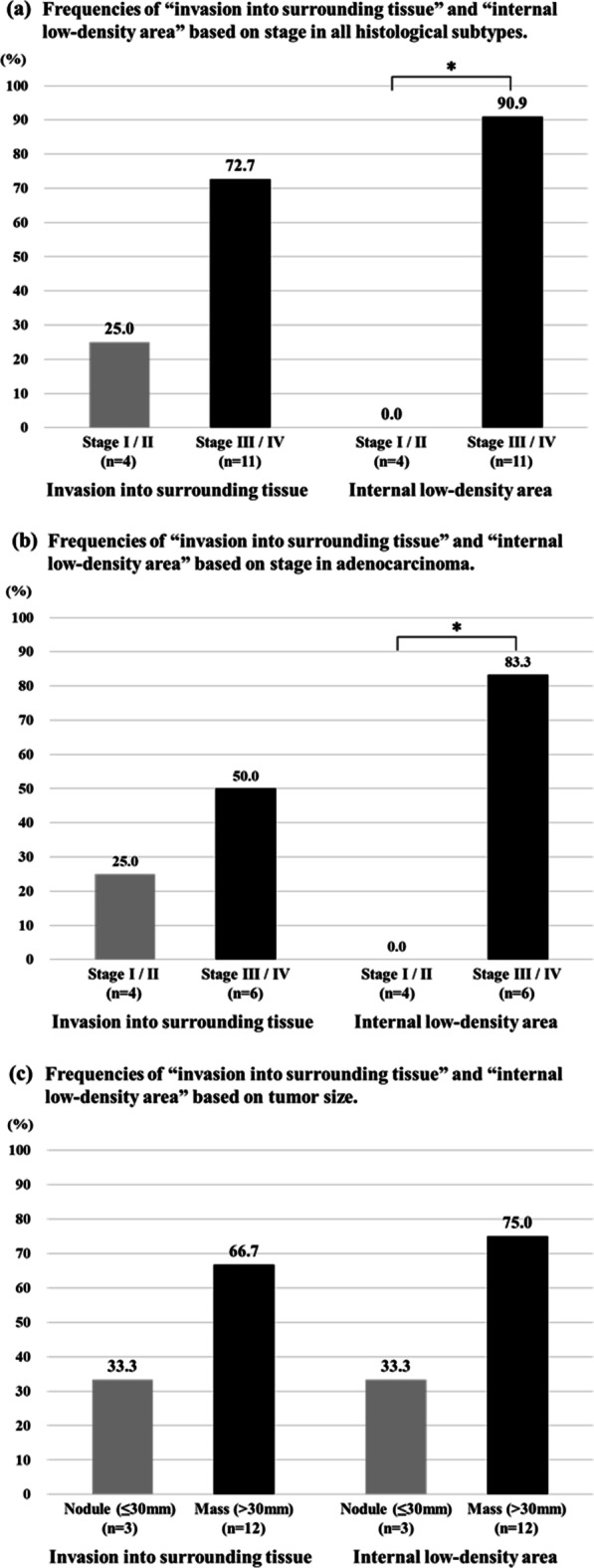
Frequencies of invasion into surrounding tissue and internal low-density areas. The frequencies of invasion into surrounding tissue and internal low-density areas were significantly higher in patients with stage III/IV NSCLC harboring METex14 skipping than in those with stage I/II disease (**a**). This tendency was also observed in patients with adenocarcinoma (n = 10) (**b**). Additionally, the frequencies of invasion into surrounding tissue and internal low-density areas were higher in masses larger than 30 mm (c). *p < 0.05, Fisher’s exact test

**Fig. 2:**
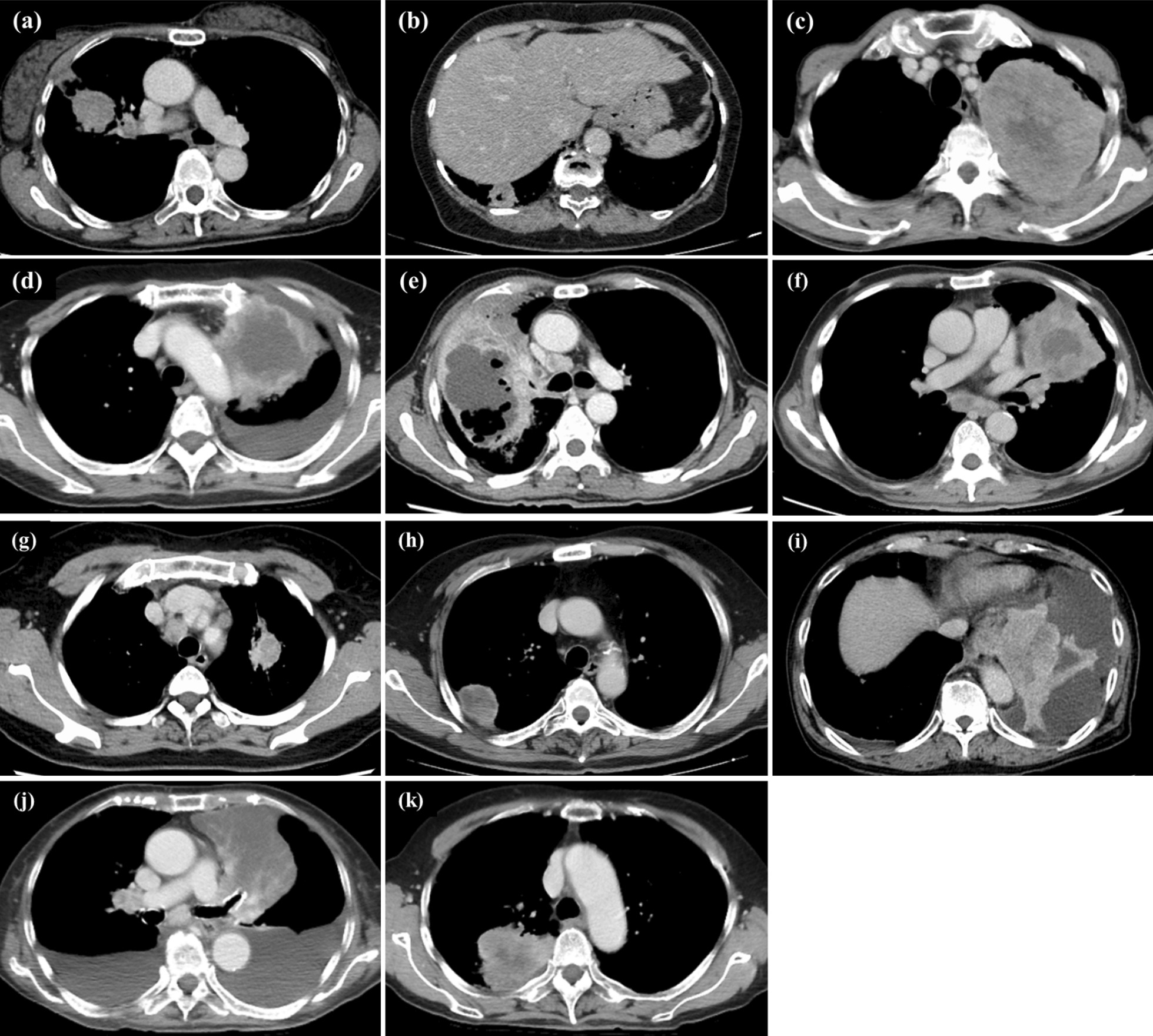
CT images of stage III/IV NSCLC with METex14 skipping. **a**–**e** are CT images of patients with stage III NSCLC, and **f**–**k** are those of patients with stage IV NSCLC. **a** does not show invasion into surrounding tissue nor internal low-density areas. **b**–**e** and **h**–**k** show both invasion into the surrounding tissue and internal low-density areas. **f** and **g** show the internal low-density areas

Subgroup analyses focusing on the tumor stage and size as well as histological subtype were performed. In stage III/IV METex14 skipping-positive NSCLC, the primary tumors invaded into surrounding tissue more frequently and there were more cases of an internal low-density areas; in 72.7% (8/11) and 90.9% (10/11) of patients, respectively (Table 2 and Fig. 1a). Figure 2 shows the CT images of the primary tumor obtained from all five patients with stage III disease and all six patients with stage IV disease. Additionally, these CT features were observed in 50.0% (3/6) and 83.3% (5/6) patients with stage III / IV adenocarcinoma, respectively (Fig. 1b, Additional file 1: Table S2). Furthermore, the tumors invaded into surrounding tissue more frequently and there were more cases of an internal low-density areas in primary tumors larger than 30 mm (8/12; 66.7% and 9/12; 75.0%, respectively) (Fig. 1c, Additional file 1: Table S3).

### CT imaging features of lymph node metastases and distant metastases

**Fig. 3:**
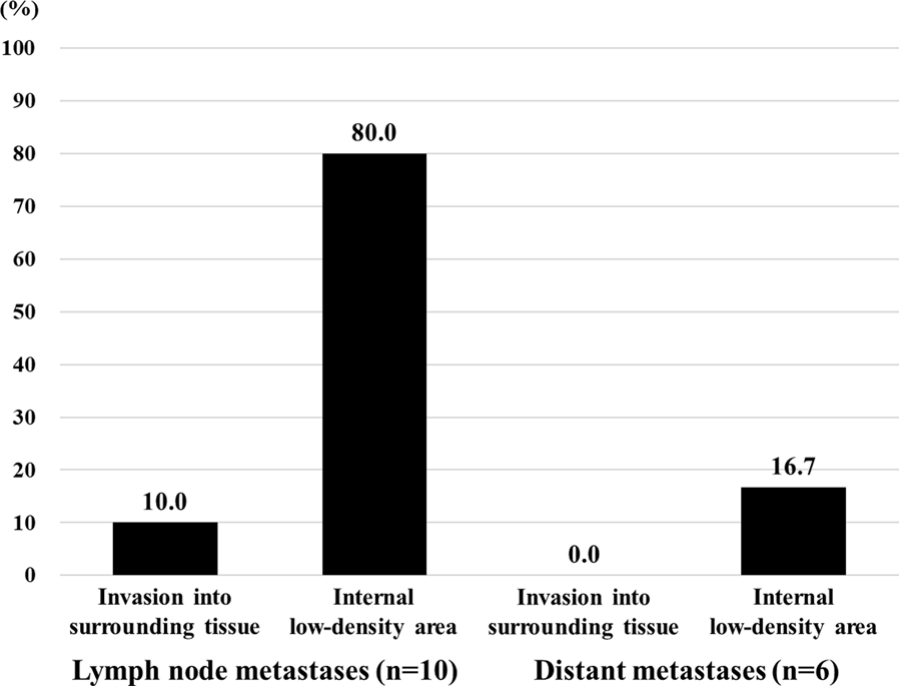
Frequencies of invasion into surrounding tissue and internal low-density areas in lymph node metastases and distant metastases

The CT imaging features of lymph node metastases and distant metastases in patients with stage III/IV NSCLC are summarized in Fig. 3 and Additional file 1: Table S4. In lymph node metastases, the presence of an internal low-density areas was frequently observed (8/10; 80.0%), while invasion into surrounding tissue was less frequently observed (1/10; 10.0%).

**Fig. 4:**
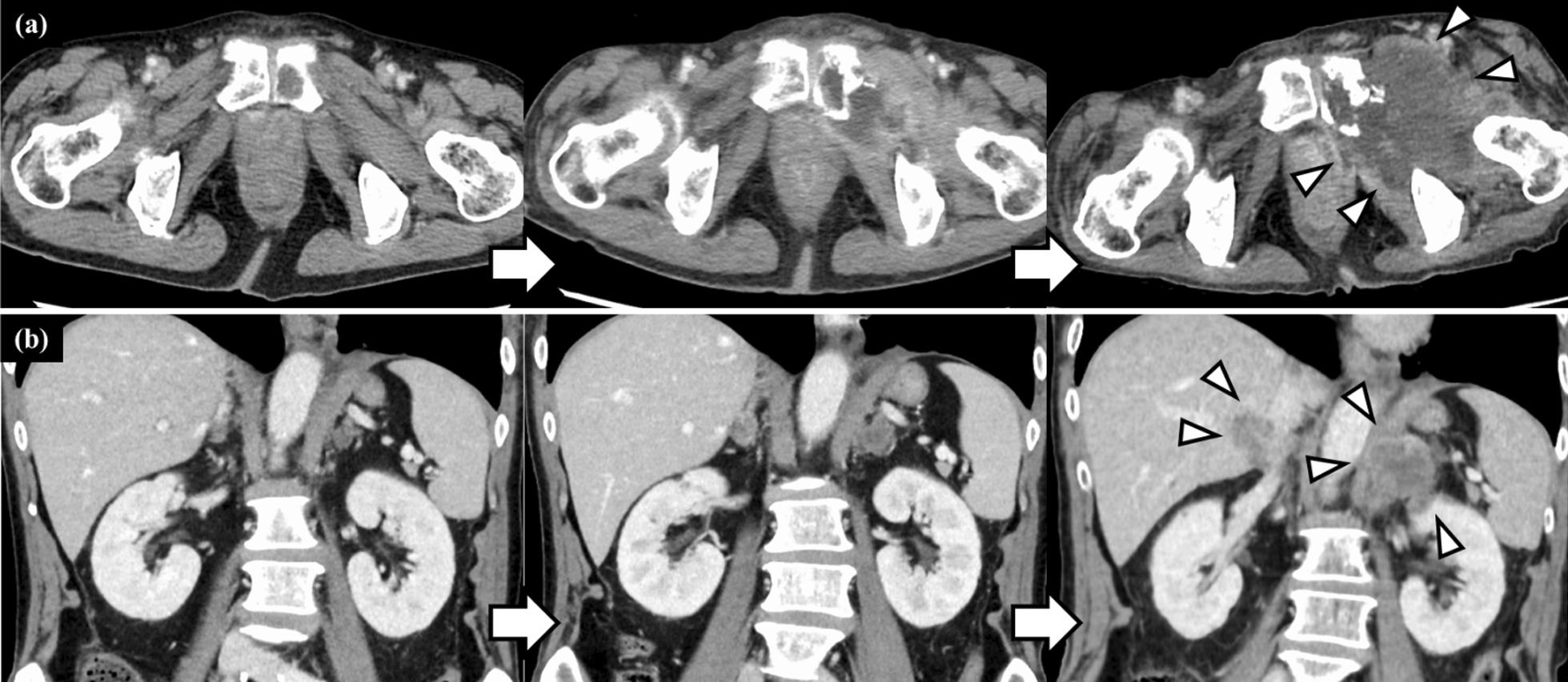
Clinical course of CT findings in distant metastases. **a** shows the clinical course of the CT findings in patient 1, whose primary tumor is shown in Fig. 2d. The left pubic bone metastasis with an internal low-density area infiltrated the surrounding muscles. **b** shows the clinical course of the CT findings in patient 2, whose primary tumor is shown in Fig. 2i. Bilateral adrenal metastases with an internal low-density area gradually infiltrated the liver, left kidney, and diaphragmatic crura

In distant metastases, neither invasion into surrounding tissue nor the presence of internal low-density areas were noted. However, in the two representative cases shown in Fig. 4, distant metastases were found to invade into surrounding tissue and possess an internal low-density area during chemotherapy with increasing metastatic tumor size. Patient 1 was a 77-year-old man with stage IIIB pleomorphic carcinoma (Fig. 2d). He underwent right upper lung lobectomy and received postoperative chemotherapy, but the tumor recurred with left pubic bone metastasis. The left pubic bone metastasis possessed an internal low-density area and gradually infiltrated the surrounding muscle (Fig. 4a). Patient 2 was a 71-year-old man with stage IVB adenocarcinoma (Fig. 2i). He received chemotherapy, and the intrathoracic lesions almost completely disappeared; however, he was diagnosed with progressive disease due to bilateral adrenal metastasis. Bilateral adrenal metastases possessed internal low-density areas and gradually infiltrated the liver, left kidney, and diaphragmatic crura (Fig. 4b).

## Discussion

Our results indicate that invasion into surrounding tissue and the presence of internal low-density areas in CT images were frequently observed in 15 patients with METex14 skipping-positive NSCLC. These CT characteristics are more obvious in patients with advanced disease. To the authors’ knowledge, this is the first study on CT characteristics of margin and internal structure of METex14 skipping-positive NSCLC tumors. On the other hand, other clinical demographics were consistent with previous research: elderly onset, a relatively high proportion of pleomorphic carcinoma and squamous cell carcinoma compared to the proportion in EGFR-mutated or ALK-rearranged NSCLC, and no significant differences in sex or smoking history [13, 15–19]. Moreover, the majority of the primary lesions appeared as a mass of 30 mm or more in the upper lobe, as found previously [13]. These consistencies support the generality of these 15 patients as METex14 skipping-positive NSCLC, and therefore invasion into surrounding tissue and presence of internal low-density areas would be valid as the characteristic features of CT findings in patients with METex14 skipping-positive NSCLC.

In the primary tumors harboring METex14 skipping, invasion into surrounding tissue was observed in 60% of cases overall and was more frequent in stage III/IV (72.7%). MET encodes a protein tyrosine kinase and regulates important cellular processes, including cell differentiation, proliferation, cell cycle, movement, and apoptosis. Hepatocyte growth factor (HGF) is a paracrine-signaling molecule secreted by mesenchymal cells that acts as a ligand for the MET receptor. METex14 skipping leads to decreased degradation of MET receptors, resulting in the activation of the MET/HGF pathway [20]. Although the association between METex14 skipping and tumor invasion into surrounding tissue observed in CT imaging is not well understood, there are two potential mechanisms. One possibility is that epithelial-to-mesenchymal transition (EMT) induced by MET signal causes tumor invasion into surrounding tissue. The activated MET/HGF pathway promotes EMT in several types of cancers [21]. EMT is associated with altered cell morphology, increased migration capacity, and high invasiveness [22]. Another possibility is that enlargement of the primary tumor related to MET signal causes invasion into surrounding tissue. In line with a previous study, the size of primary tumors > 30 mm at diagnosis is a dominant feature in patients with METex14 skipping-positive NSCLC [13]. We also found that invasion into surrounding tissue was observed more frequently when the primary tumor was larger than 30 mm. Additionally, in distant metastases, invasion into the surrounding tissue became obvious with increasing metastatic tumor size. These data suggest that aggravating MET signaling promoted by METex14 skipping accelerates tumor invasion into the surrounding tissue by inducing EMT and/or tumor enlargement.

The frequency of internal low-density areas in the primary tumor and lymph node metastasis was high in METex14 skipping-positive NSCLC (9/15; 60.0% and 8/10; 80.0%, respectively). This trend was also observed when only adenocarcinomas were analyzed. CT findings of internal low-density areas as well as invasion into surrounding tissue are well known as the typical features of pleomorphic carcinoma [23]. Pleomorphic carcinoma is classified as a sarcomatoid carcinoma and it is defined as poorly differentiated NSCLC that contains at least 10% spindle and/or giant cells, or a carcinoma consisting only of spindle and giant cells [24]. Approximately 20%–30% of pulmonary sarcomatoid carcinomas harbor METex14 alterations, although METex14 alterations are found in 2%–3% of adenocarcinomas [4]. In contrast, adenocarcinomas with METex14 skipping frequently contain giant cells with nuclear pleomorphisms [25]. These data suggest that sarcomatoid carcinoma components such as highly pleomorphic tumor cells with multiple large, irregularly lobulated nuclei may be characteristic of METex14 skipping [25]. Thus, the CT findings of METex14 skipping-positive NSCLC may resemble those of pleomorphic carcinoma represented by invasion into surrounding tissue and the presence of internal low-density areas.

The CT features of METex14 skipping-positive NSCLC might be useful when a tumor tissue-based multiplex assay to detect multiple driver oncogene mutations fails. For example, we can currently search for multiple driver oncogene mutations with the Oncomine Dx Target Test Multi‐CDx System at cancer diagnosis, but it requires a sufficient amount of tumor sample. Because of the small size of tumor samples, the failure rate of mutation detection with such a multiplex assays is approximately 25% when the sample is obtained by bronchoscopy [26–28]. METex14 skipping is also detected in ctDNA extracted from blood samples with ArcherMET. Importantly, the CT features of METex14 skipping-positive NSCLC were different from those of EGFR mutation-positive NSCLC and those of ALK rearranged-positive NSCLC evaluated in previous studies. For example, previous studies have demonstrated that EGFR mutation-positive NSCLC did not show primary tumors larger than 30 mm in size with internal low-density areas frequently (10%), while pleural invasion was reported to be almost 50% [29–31]. Additionally, internal low-density areas and invasion into surrounding tissue of primary tumors are infrequent in ALK-rearranged-positive NSCLC (10% and 10–20%, respectively) [26, 27]. Therefore, when internal low-density areas are present and primary tumors invade into surrounding tissue as observed in CT images and the tissue sample is not large enough to be submitted to tumor tissue-based test targeting multiple driver oncogene mutations, the investigation of METex14 skipping by single-plex assay such as ArcherMET may take priority over that of EGFR mutation and/or ALK rearrangement.

Our study had several limitations. Because of the rarity, the sample size was relatively small, and a comparative analysis elucidating the difference in CT findings between NSCLC with METex14 skipping and those with other rare driver oncogenes, such as ROS1, BRAF and RET, could not be performed. Second, the association between CT findings and histological subtypes was mainly evaluated based on small tumor samples obtained by bronchoscopy, although the histological diagnosis of NSCLC, especially pleomorphic carcinoma, is influenced by the proportion of spindle and/or giant cells. Third, contrast-enhanced CT was not performed in three of four patients with stage I/II NSCLC; thus, the prevalence of internal low-density areas may have been underestimated in these patients. Additionally, the protocol of contrast-enhanced CT scanning was not unified because of the retrospective design of this multicentor study. Finally, METex14 skipping in seven patients was investigated and detected only by the Oncomine Dx Target Test Multi-CDx System, which has a false-positive rate of approximately 30% for METex14 skipping [32]. Therefore, further studies with a larger sample size are needed to elucidate the association between METex14 skipping and CT findings with consideration of histological subtypes and to perform unified investigation of METex14 skipping.

## Conclusion

METex14 skipping-positive NSCLC tumors tend to invade into surrounding tissue and possess internal low-density areas observed on CT images. These CT findings might be characteristic of METex14 skipping-positive NSCLC.

## Supplementary Information

**Additional file 1**.** Table S1**. Summary of clinicopathological data and CT findings of 15 patients with METex14 skipping-positive NSCLC.** Table S2**. CT findings of the primary tumor in adenocarcinoma.** Table S3**. CT imaging features of the primary tumor compared to the tumor size.** Table S4**. CT findings of lymph nodes and distant metastases. (a) CT findings of lymph node metastases in patients with stage III/IV NSCLC (n=10). (b) Patterns of distant metastasis in patients with stage IV NSCLC (n=6). (c) CT findings of distant metastases in patients with stage IV NSCLC (n=6).** Figure S1**. CT images of stage I/II NSCLC with METex14 skipping.

## Data Availability

The datasets used and/or analyzed during the current study are available from the corresponding author on reasonable request.

## Abbreviations

NSCLC: Non-small cell lung cancer
METex14: Mesenchymal-epithelial transition exon14
TKIs: Tyrosine kinase inhibitors
CT: Computed tomography
EGFR: Epidermal growth factor receptor
GGO: Ground-glass opacity
ALK: Anaplastic lymphoma kinase
HGF: Hepatocyte growth factor
EMT: Epithelial-to-mesenchymal transition
