# Supplementary material for: Characteristic computed tomography features in mesenchymal-epithelial transition exon14 skipping-positive non-small cell lung cancer
Source: BMC Pulm Med. 2022 Jun 30;22:260. doi: 10.1186/s12890-022-02037-4 (PMC9245203; doi:10.1186/s12890-022-02037-4)
Supplement: Supplementary file 1 — Additional file 1. Table S1. Summary of clinicopathological data and CT findings of 15 patients with METex14 skipping-positive NSCLC. Table S2. CT findings of the primary tumor in adenocarcinoma. Table S3. CT imaging features of the primary tumor compared to the tumor size. Table S4. CT findings of lymph nodes and distant metastases. (a) CT findings of lymph node metastases in patients with stage III/IV NSCLC (n=10). (b) Patterns of distant metastasis in patients with stage IV NSCLC (n=6). (c) CT findings of distant metastases in patients with stage IV NSCLC (n=6). Figure S1. CT images of stage I/II NSCLC with METex14 skipping. [file 12890_2022_2037_MOESM1_ESM.docx]

Table S1. Summary of clinicopathological data and CT findings of 15 patients with METex14 skipping-positive NSCLC.

|  | Sex | Age, years | Smoking History | Histological type | Molecular Testing | Stage | Primary site | Primary tumor | |  | Lymphadenopathy | |  | Distant metastases | |
| --- | --- | --- | --- | --- | --- | --- | --- | --- | --- | --- | --- | --- | --- | --- | --- |
|  |  |  |  |  |  |  |  | Invasion into surrounding tissue | Internal  low-density area |  | Invasion into surrounding tissue | Internal  low-density area |  | Invasion into surrounding tissue | Internal  low-density area |
| Figure S1 (a) | Female | 89 | never | Adeno | Oncomine | ⅠA2 | Left upper lobe | (-) | (-) |  | NA | NA |  | NA | NA |
| Figure S1 (b) | Male | 84 | former | Adeno | ArcherMET | ⅠA3 | Right upper  lobe | (-) | (-) |  | NA | NA |  | NA | NA |
| Figure S1 (c) | Female | 86 | never | Adeno | ArcherMET | ⅠA3 | Right upper  lobe | (-) | (-) |  | NA | NA |  | NA | NA |
| Figure S1 (d) | Male | 71 | former | Adeno | ArcherMET | ⅡB | Left lower segment | (+) | (-) |  | NA | NA |  | NA | NA |
| Figure 2 (a) | Female | 71 | former | Adeno | Oncomine | ⅢA | Right upper lobe | (-) | (-) |  | (-) | (-) |  | NA | NA |
| Figure 2 (b) | Female | 74 | former | Adeno | Oncomine | ⅢA | Right lower  Lobe | (+) | (+) |  | (-) | (+) |  | NA | NA |
| Figure 2 (c) | Male | 75 | former | Pleomorphic | ArcherMET | ⅢA | Left upper  Lobe | (+) | (+) |  | NA | NA |  | NA | NA |
| Figure 2 (d) | Female | 79 | never | Squamous | ArcherMET | ⅢA | Left upper segment | (+) | (+) |  | (-) | (+) |  | NA | NA |
| Figure 2 (e) | Male | 77 | former | Pleomorphic | Oncomine | ⅢB | Right upper segment | (+) | (+) |  | (-) | (+) |  | NA | NA |
| Figure 2 (f) | Male | 70 | former | Adeno | Oncomine | ⅣA | Left upper segment | (-) | (+) |  | (-) | (+) |  | (-) | (-) |
| Figure 2 (g) | Female | 67 | never | Adeno | ArcherMET | ⅣA | Left upper  lobe | (-) | (+) |  | (-) | (+) |  | (-) | (-) |
| Figure 2 (h) | Male | 63 | former | Adeno | ArcherMET | ⅣB | Right upper  lobe | (+) | (+) |  | (-) | (+) |  | (-) | (-) |
| Figure 2 (i) | Male | 71 | former | Adeno | ArcherMET | ⅣB | Left lower lobe | (+) | (+) |  | (+) | (+) |  | (-) | (-) |
| Figure 2 (j) | Female | 94 | never | Pleomorphic | Oncomine | ⅣB | Left upper  lobe | (+) | (+) |  | (-) | (+) |  | (-) | (-) |
| Figure 2 (k) | Female | 79 | never | Squamous | Oncomine | ⅣB | Right upper segment | (+) | (+) |  | (-) | (-) |  | (-) | (+) |

Abbreviations: Oncomine, Oncomine Dx Target Test Multi‐CDx System; NA, not available.

|  | All patients | Stage Ⅰ / Ⅱ | Stage Ⅲ / Ⅳ |
| --- | --- | --- | --- |
| Subjects, n | 10 | 4 | 6 |
| Size |  |  |  |
| Median (mm) | 35.0 (26.8-50.0) | 31.5 (22.0-42.5) | 37.5 (29-68.8) |
| Mass (>30mm), n (%) | 7 (70.0) | 2 (50.0) | 5 (83.3) |
| Nodule (≤30mm), n (%) | 3 (30.0) | 2 (50.0) | 1 (16.7) |
| Lobar location |  |  |  |
| Upper lobe/Upper segment, n (%) | 7 (70.0) | 3 (75.0) | 4 (66.7) |
| Middle lobe/Lingular segment, n (%) | 0 (0.0) | 0 (0.0) | 0 (0.0) |
| Lower lobe, n (%) | 3 (30.0) | 1 (25.0) | 2 (33.3) |
| Margin |  |  |  |
| Invasion into surrounding tissue, n (%) | 4 (40.0) | 1 (25.0) | 3 (50.0) |
| Lobulation, n (%) | 1 (10.0) | 0 (0.0) | 1 (16.7) |
| Pleural indentation, n (%) | 3 (30.0) | 1 (25.0) | 2 (33.3) |
| Spicula, n (%) | 3 (30.0) | 1 (25.0) | 2 (33.3) |
| Ground-glass opacity, n (%) | 1 (10.0) | 1 (25.0) | 0 (0.0) |
| Internal structure |  |  |  |
| Air bronchograms, n (%) | 2 (20.0) | 1 (25.0) | 1 (16.7) |
| Cavitation, n (%) | 1 (10.0) | 0 (0.0) | 1 (16.7) |
| Internal low-density area, n (%) | 5 (50.0) | 0 (0.0) | 5 (83.3) |

Table S2. CT findings of the primary tumor in adenocarcinoma.

Data are presented as medians and interquartile ranges.

|  | All | Nodule (≤30mm) | Mass (>30mm) |
| --- | --- | --- | --- |
| Subjects, n | 15 | 3 | 12 |
| Lobar location |  |  |  |
| Upper lobe/Upper segment, n (%) | 12 (80.0) | 2 (66.7) | 10 (83.3) |
| Middle lobe/Lingular segment, n (%) | 0 (0.0) | 0 (0.0) | 0 (0.0) |
| Lower lobe, n (%) | 3 (20.0) | 1 (33.3) | 2 (16.7) |
| Margin |  |  |  |
| Invasion into surrounding tissue, n (%) | 9 (60.0) | 1 (33.3) | 8 (66.7) |
| Lobulation, n (%) | 2 (13.3) | 0 (0.0) | 2 (16.7) |
| Pleural indentation, n (%) | 3 (20.0) | 1 (33.3) | 2 (16.7) |
| Spicula, n (%) | 3 (20.0) | 0 (0.0) | 3 (25.0) |
| Ground-glass opacity, n (%) | 1 (6.7) | 1 (33.3) | 0 (0.0) |
| Internal structure |  |  |  |
| Air bronchograms, n (%) | 2 (13.3) | 2 (66.7) | 0 (0.0) |
| Cavitation, n (%) | 2 (13.3) | 1 (33.3) | 1 (8.3) |
| Internal low-density area, n (%) | 10 (66.7) | 1 (33.3) | 9 (75.0) |

Table S3. CT imaging features of the primary tumor compared to the tumor size.

Table S4. CT findings of lymph nodes and distant metastases.

(a) CT findings of lymph node metastases in patients with stage III/IV NSCLC (n=10).

|  | n | (%) |
| --- | --- | --- |
| Margin |  |  |
| Invasion into surrounding tissue | 1 | 10.0 |
| Internal structure |  |  |
| Cavitation | 0 | 0.0 |
| Internal low-density area | 8 | 80.0 |

(b) Patterns of distant metastasis in patients with stage IV NSCLC (n=6).

|  | n | (%) |
| --- | --- | --- |
| Intrathoracic Metastasis |  |  |
| Lung | 3 | 50.0 |
| Lymphangitic carcinomatosis | 0 | 0.0 |
| Pleural | 3 | 50.0 |
| Pericardial | 0 | 0.0 |
| Extrathoracic Metastasis |  |  |
| Adrenal | 2 | 33.3 |
| Liver | 1 | 16.7 |
| Gastric | 0 | 0.0 |
| Splenic | 0 | 0.0 |
| Bone | 1 | 16.7 |
| Brain | 2 | 33.3 |
| Soft tissue | 0 | 0.0 |

(c) CT findings of distant metastases in patients with stage IV NSCLC (n=6).

|  | n | (%) |
| --- | --- | --- |
| Margin |  |  |
| Invasion into surrounding tissue | 0 | 0.0 |
| Internal structure |  |  |
| Cavitation | 0 | 0.0 |
| Internal low-density area | 1 | 16.7 |


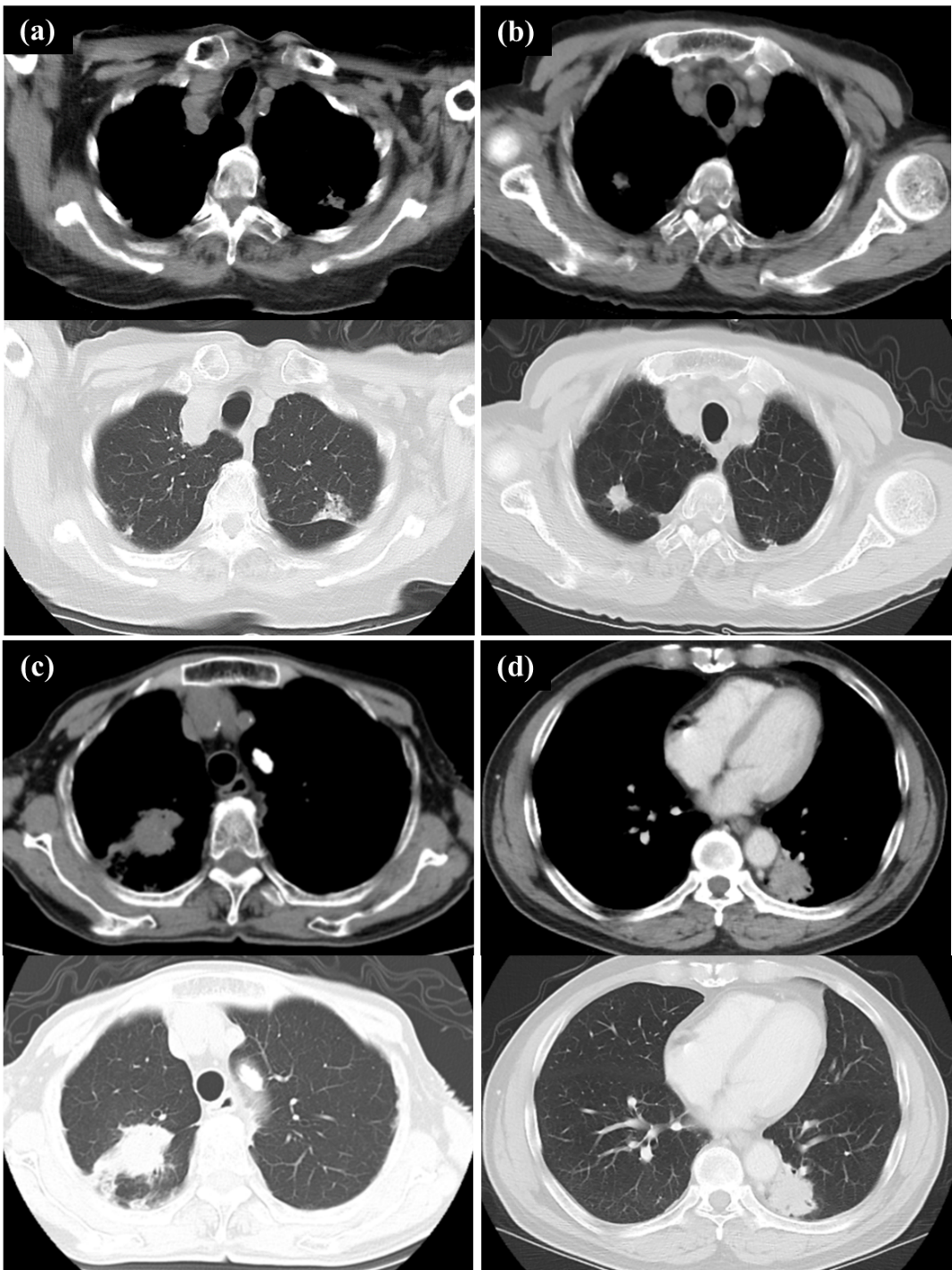


Figure S1. CT images of stage I/II NSCLC with METex14 skipping.
